# Animal Assisted Therapy (AAT) Program As a Useful Adjunct to Conventional Psychosocial Rehabilitation for Patients with Schizophrenia: Results of a Small-scale Randomized Controlled Trial

**DOI:** 10.3389/fpsyg.2016.00631

**Published:** 2016-05-06

**Authors:** Paula Calvo, Joan R. Fortuny, Sergio Guzmán, Cristina Macías, Jonathan Bowen, María L. García, Olivia Orejas, Ferran Molins, Asta Tvarijonaviciute, José J. Cerón, Antoni Bulbena, Jaume Fatjó

**Affiliations:** ^1^Chair Affinity Foundation Animals and Health, Department of Psychiatry and Forensic Medicine, Universitat Autònoma de BarcelonaBellaterra, Spain; ^2^Hospital del Mar Medical Research InstituteBarcelona, Spain; ^3^Centres Assistencials Emili Mira, Institut de Neuropsiquiatria i Addiccions, Parc de Salut MarSanta Coloma de Gramenet, Spain; ^4^Queen Mother Hospital for Small Animals, The Royal Veterinary CollegeHertfordshire, UK; ^5^Interlab-UMU, Campus de Excelencia Mare Nostrum, Universidad de MurciaMurcia, Spain; ^6^Department of Medicine and Animal Surgery, Universitat Autònoma de BarcelonaBellaterra, Spain

**Keywords:** animal-assisted therapy, psychosocial rehabilitation, adherence to treatment, schizophrenia, PANSS, EuroQol-5 dimensions, salivary cortisol, salivary alpha-amylase

## Abstract

Currently, one of the main objectives of human–animal interaction research is to demonstrate the benefits of animal assisted therapy (AAT) for specific profiles of patients or participants. The aim of this study is to assess the effect of an AAT program as an adjunct to a conventional 6–month psychosocial rehabilitation program for people with schizophrenia. Our hypothesis is that the inclusion of AAT into psychosocial rehabilitation would contribute positively to the impact of the overall program on symptomology and quality of life, and that AAT would be a positive experience for patients. To test these hypotheses, we compared pre–program with post–program scores for the Positive and Negative Syndrome Scale (PANSS) and the EuroQoL-5 dimensions questionnaire (EuroQol-5D), pre–session with post–session salivary cortisol and alpha–amylase for the last four AAT sessions, and adherence rates between different elements of the program. We conducted a randomized, controlled study in a psychiatric care center in Spain. Twenty–two institutionalized patients with chronic schizophrenia completed the 6–month rehabilitation program, which included individual psychotherapy, group therapy, a functional program (intended to improve daily functioning), a community program (intended to facilitate community reintegration) and a family program. Each member of the control group (n = 8) participated in one activity from a range of therapeutic activities that were part of the functional program. In place of this functional program activity, the AAT–treatment group (n = 14) participated in twice–weekly 1–h sessions of AAT. All participants received the same weekly total number of hours of rehabilitation. At the end of the program, both groups (control and AAT–treatment) showed significant improvements in positive and overall symptomatology, as measured with PANSS, but only the AAT–treatment group showed a significant improvement in negative symptomatology. Adherence to the AAT-treatment was significantly higher than overall adherence to the control group’s functional rehabilitation activities. Cortisol level was significantly reduced after participating in an AAT session, which could indicate that interaction with the therapy dogs reduced stress. In conclusion, the results of this small-scale RCT suggest that AAT could be considered a useful adjunct to conventional psychosocial rehabilitation for people with schizophrenia.

## Introduction

Interactions with companion animals appear to have positive effects on physiological, psychological, and social aspects of human wellbeing (Fine, 2010). Animal assisted therapies (AAT) seem to produce therapeutic benefits in different kinds of patients, from those with physical ailments, such as cardiovascular disease, to those with mental disorders ranging from dementia to depression (Pedersen et al., 2011) and schizophrenia (Barak et al., 2001). It has been suggested that AAT might help to develop the therapeutic relationship between patients and healthcare professionals, and could improve the therapeutic atmosphere (Fine, 2010; Julius et al., 2013); animals in AAT can act as social facilitators, social modulators, and amplifiers of emotional reactivity (Fine, 2010).

However, scientific evidence for the benefits of AAT is still very limited (Nimer and Lundahl, 2007; Kamioka et al., 2014), partially due to intrinsic difficulties of performing research with AAT (Nimer and Lundahl, 2007; Kamioka et al., 2014). Typical methodological limitations of AAT include: small sample size, difficulties of blinding, lack of an adequate control group, selection bias due to including only participants who like animals, lack of physiological evaluation, short program duration and the limited number of professionals and animals that currently participate in AAT. Some of these limitations are very difficult to overcome, because of the nature of AAT interventions. For example, in AAT, it is very difficult to find a comparable therapeutic activity for the control group, and it is impossible to blind for the presence of the animal. Since AAT is still considered an alternative therapeutic approach, very few resources are dedicated to it within the health system (Kaplan and Sadock, 1989). As a consequence of these limitations it is important to compile studies with partial evidence for AAT efficacy and applicability (Fine, 2010) and to improve and standardize research methodologies (Kamioka et al., 2014).

Recent reviews of AAT research indicate that mental health disorders are a good target for AAT interventions (Nimer and Lundahl, 2007; Villalta-Gil and Ochoa, 2007; Rossetti and King, 2010; Kamioka et al., 2014). Some studies have shown that AAT programs could benefit patients being treated for schizophrenia (Kovács et al., 2004, 2006; Nathans-Barel et al., 2005; Chu et al., 2009). Suggested benefits include effects on self-esteem, self-determination, positive symptomatology, emotional symptomatology, anhedonia, and daily functioning (Nathans-Barel et al., 2005; Villalta-Gil and Ochoa, 2007; Villalta-Gil et al., 2009; Kamioka et al., 2014).

The aim of this study was to assess the effect of an AAT program as an adjunct to conventional psychosocial rehabilitation for people with schizophrenia.

Based on the hypothesis that inclusion of AAT in a rehabilitation program would have a beneficial effect, our study had three objectives; to analyze the impact on symptomatology and quality of life, to evaluate the patient’s experience of the AAT sessions, and to assess stress relief during the AAT sessions. For the first objective, the measures used were the Positive and Negative Syndrome Scale (PANSS; Kay et al., 1989; Peralta and Cuesta, 1994), and EuroQoL-5 Dimensions questionnaire (EQ-5D; Bobes et al., 2005). For the second objective, we used adherence (proportion of programmed sessions that a patient attended). Adherence was used as an indicator of the relative appeal of the AAT sessions, by comparing adherence for the AAT sessions with combined adherence for the functional program attended by the control group. For the last objective, since stress management is one of the main objectives for the treatment of inpatients with mental disorders (Klainin-Yobas et al., 2015), we evaluated the stress-relieving aspect of the sessions by making a pre- versus post-session comparison of values for salivary cortisol and alpha-amylase for the last four AAT sessions. To our knowledge, previous research on the effects of AAT for patients with schizophrenia has not included the combination of these three different types of objectives (and the associated measures).

Our general objective was to present evidence that was different and complementary to existing research and to identify interesting target measures, such as adherence to treatment and physiological measures, that could be used for future research.

## Materials and Methods

### Study Design

The study was a randomized, controlled trial (RCT).

In this study, primary outcomes for all participants were changes in symptomatology (measured with PANSS) and changes in quality of life (measured with EQ-5D). Secondary outcomes of this study consisted of adherence to AAT sessions (AAT-treatment group) versus adherence to other activities of functional rehabilitation (control group), and changes in salivary cortisol and alpha-amylase during AAT sessions, as a measure of stress relief (AAT-treatment group only).

Patients were randomly assigned to the control or AAT-treatment group.

The laboratory technicians who analyzed the saliva samples were only given the patients’ ID numbers, and were blinded to whether patients were in the control or AAT-treatment group. For practical reasons and for issues relating to the availability of resources and personnel, the rest of the process of the study could not be blinded. It was not possible for patients to be blinded to the presence of dogs, and only one hospital neuropsychologist was able to participate in the study (in charge of all of the pre-treatment and post-treatment evaluations of the study, and follow-up of all of the patients). A single researcher not only carried out the collection of the data and saliva samples, but also acted as a guide for the therapy dogs during the AAT sessions.

### Sample

The study was conducted in a public psychiatric hospital within an urban area of Spain. In order to avoid the confounding effects of environmental variation, only patients from the same unit were included (MILLE: Long and medium-stay unit). All eligible patients from the MILLE unit who fulfilled the following criteria were included:

• Diagnosis of schizophrenia, according to the Revised forth edition of the Diagnostic and Statistical Manual of Mental Disorders (DSM-IV-TR; American Psychiatric Association, 2000).
• Enrolled in a psychosocial rehabilitation process.
• With a projected minimum hospitalization term of 6 months.

A set of exclusion criteria was also applied, which included:

• Compromised mobility.
• Presence of allergies to animals.
• Rejection of contact with companion animals.
• Confirmed diagnosis of a coagulopathy.

These inclusion and exclusion criteria were adapted from previous AAT protocols (Barak et al., 2001; Kovács et al., 2004; Nathans-Barel et al., 2005; Villalta-Gil et al., 2009; Fine, 2010; Lang et al., 2010). All patients in the unit who met the criteria were included in the study.

Twenty-four adult patients (Mean age = 47.8 years of age; *SD* = 6.7) fulfilled the requirements and were included in the study. The patients’ mean age at diagnosis of schizophrenia was 20.5 years of age (*SD* = 5.0). The patients’ mean scores for PANSS were: 43.8 (*SD* = 12.3) for General PANSS, 24 (*SD* = 6.6) for Negative PANSS and 20.6 (*SD* = 6.6) for Positive PANSS. The EQ-5D total score mean was 1.8 (*SD* = 1.5). See **Table 1** for an overview of all of the characteristics of the sample population.

**Table 1:** Characteristics of the sample.

Patient	Group	Age	Age of onset	Gender	PANSS General	PANSS Negative	PANSS Positive	EQ-5D total	Psychotropic medication	Other medication
1	T	54	17	Man	31	25	11	0	Aripripazol, levomepromazine	Pravastatin, repaglinide, pantoprazole
2	T	66	20	Woman	38	18	22	3	Clozapine, venlafaxine	Pantoprazole, lactulose
3	T	37	21	Man	32	17	9	1	Clozapine, amisulpride, lorazepam, pregabalin	–
4	T	58	22	Man	46	36	24	2	Fluphenazine, levomepromazine, biperiden	–
5	T	52	26	Man	50	32	23	1	Clozapine, sodium valproate	Metformin
6	T	47	20	Man	38	19	13	1	Zuclopenthixol, biperiden	Atorvastatin
7	T	41	15	Man	77	39	28	3	Levomepromazine, periciazine, paroxetine, biperiden	–
8	T	33	23	Woman	31	12	15	0	Zuclopenthixol, clonazepam, sodium valproate, biperiden	Gemfibrozil
9	T	35	24	Man	46	25	18	2	Clozapine, fluphenazine, pimozide, clotiapine	–
10	T	47	21	Man	53	29	25	1	Fluphenazine, risperidone, biperiden	Lactulose
11	T	54	15	Woman	37	29	17	1	Risperidone, zuclopenthixol, sodium valproate	–
12	T	44	22	Woman	37	22	27	3	Clozapine, risperidone	Atorvastatin, salmeterol
13	T	50	17	Man	43	24	17	7	Lithium carbonate, clozapine, methylphenidate, sertraline, topiramate, biperiden	Monohydrate lactitol
14	T	55	39	Man	61	27	15	2	Olanzapine, paroxetine, dipotassium cloracepate, biperiden	Hydroxyzine, loratadine
15	T	43	21	Man	47	30	30	2	Fluphenazine, risperidone, sodium valproate	–
16	T	45	19	Man	42	18	34	2	Clozapine, diazepam, sodium valproate	Pantoprazole, lactulose, gemfibrozil
17	C	48	18	Woman	45	26	18	4	Clozapine	–
18	C	47	16	Man	50	20	14	1	Haloperidol, dipotassium clorazepate, biperiden	Lactulose
19	C	53	21	Man	46	16	25	2	Risperidone, quetiapine, sodium valproate, clonazepam	Atenolol, pravastatin, pantoprazole, metformin
20	C	59	25	Man	44	22	21	2	Clozapine, gabapentin, biperiden	–
21	C	51	16	Man	61	26	28	0	Zuclopenthixol, ziprasidone, sodium valproate, biperiden	–
22	C	50	20	Man	15	22	12	0	clozapine	Enalapril
23	C	44	18	Woman	48	15	22	2	Zuclopenthixol, levomepromazine, clotiapine	–
24	C	50	16	Woman	32	27	26	1	Olanzapine, levomepromazine, sodium valproate, phenytoin	–
**MEAN (*SD*)**	**–**	**47.8 (6.7)**	**20.5 (5.0)**	**–**	**43.7 (12.3)**	**20.6 (6.6)**	**24 (6.6)**	**1.8 (1.5)**	**–**	–

**FIGURE 1:**
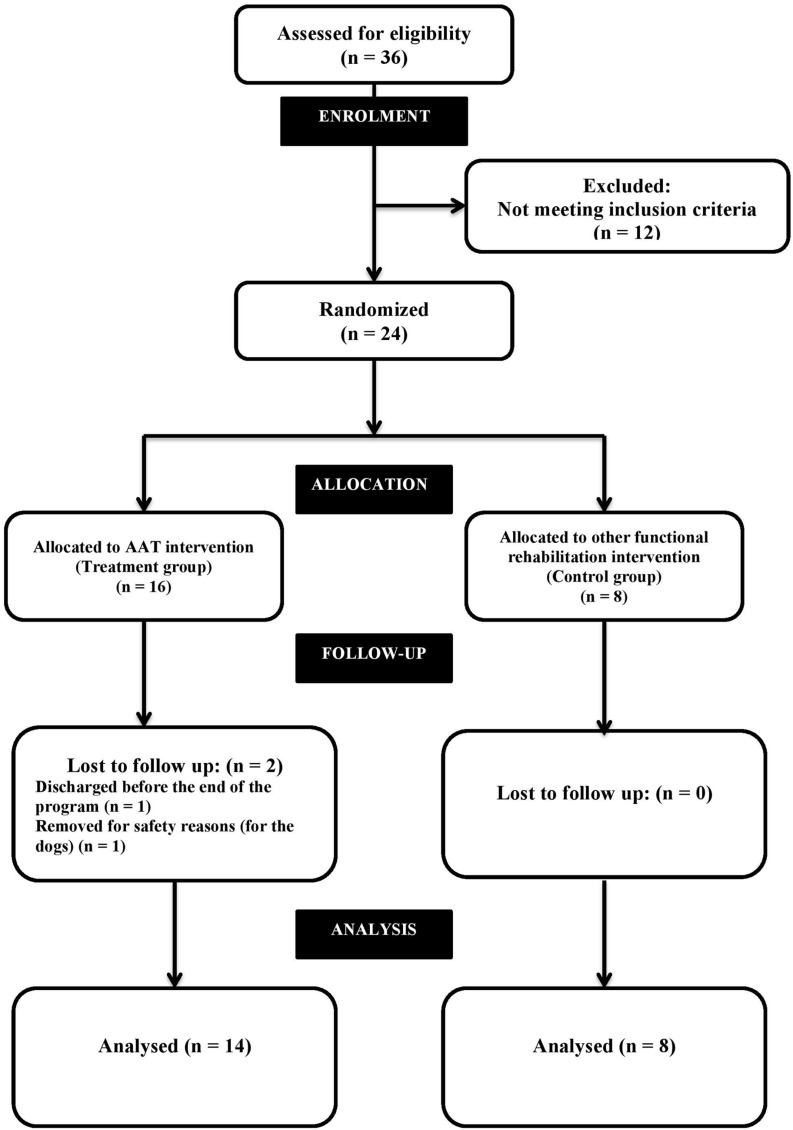
**Shows the modified CONSORT flow diagram for individual RCTs of non-pharmacologic treatment (Boutron et al., 2008; Schulz et al., 2010) applied to this study**.

The 24 patients who met the inclusion criteria were randomly assigned to three groups, with eight patients in each group (AAT-treatment groups A and B, and a control group C) (See **Figure 1**). Given the length of the study (6 months), a high drop out rate was expected. Other authors recommend that group size is kept small for AAT sessions (Kovács et al., 2004; Nathans-Barel et al., 2005; Chu et al., 2009; Fine, 2010). To comply with this recommendation, the 16 patients who were to be given AAT were randomly allocated to one of two small therapy groups (eight people in each). There were no differences in the characteristics of these groups, or in the AAT-therapy they received. In the analysis, data from patients in both therapy groups (A and B) was therefore combined into a single group.

Five therapy dogs that had previously been assessed and trained, and had experience of participation in AAT work were used for the study. There is no official dog therapy certification in Spain. A thorough physical and behavioral examination of each dog was performed by a panel of three board-certified specialists in veterinary behavioral medicine. This examination included the Ethotest (Lucidi et al., 2005), a test designed to identify suitable therapy dogs, and the C-BARQ (Hsu and Serpell, 2003), a questionnaire for measuring behavior and temperament traits in dogs.

### Interventions

The study took place between October 2012 and May 2013. At the psychiatric hospital where the study was conducted, the global psychosocial rehabilitation process consisted of five types of programs: individual psychotherapy, group therapy program, functional program (to improve daily functioning), community program (with social reintegration objectives), and family program. From Monday to Friday every week, all patients treated in this global psychosocial rehabilitation process had to participate in all five types of program.

Patients in all groups participated in the same total weekly number of hours of activity within the psychosocial rehabilitation process. For the AAT-therapy groups (A and B) the AAT program was one of these activities. The AAT program consisted of 6-months of twice-weekly 1-h sessions (Tuesday and Friday), so that each patient attended a total of 40 AAT sessions (taking into account public holidays). Control group patients attended the same number of sessions in the functional program.

The AAT-treatment involved three types of sessions:

(a) Sessions to develop the emotional bond between participants and dogs: The participants were taught to handle and take care of the dogs correctly. In this type of session, concepts of animal welfare and responsible ownership were explained and practiced.
(b) Sessions involving walking the dogs: During the first half of the program, the dogs were walked in a large natural park, so that the patients could learn to walk the dogs in a calm and controlled manner. For the rest of the program, the participants walked the dogs in the city, where they could experience dog-walking in a social context that is typical of that which is experienced by dog owners.
(c) Sessions to train and play with dogs: Patients learned to give instructions to the dogs and train them using positive reinforcement training techniques.

During an AAT session 4 of the 5 therapy dogs were always present to interact with the patients. At the beginning of each session, participants were asked to work in pairs. Each working pair was assigned a dog, which they worked with for the remaining hour of the session. During the program there was a rotation between the three types of sessions (emotional bonding, dog walking, and dog training with play).

Each patient in the control group was assigned to a single activity from the functional program on the basis of their therapist’s criteria, but taking into account the individual’s preferences. The choice was between art therapy, group sports (football or basketball), dynamic psycho-stimulation, and gymnastics. These activities were organized so that they closely matched certain important characteristics of the AAT program:

• They were conducted outside the hospital unit where the patients were resident.
• They all involved a similar element of group work.
• Group sizes were small (similar to the AAT sessions).
• Patients were accompanied and supervised off-site by a mental health professional (nurse or similar).
• The activities continued throughout the period of trial (they were unaffected by season).
• The sessions were twice-weekly and of 1-h duration.

The difference between functional program activities and the AAT sessions was, as far as was possible, restricted to content.

### Instruments

To compare evolution in psychiatric symptoms between AAT-treatment and control patients during the 6-month duration of the program, we used the previously validated Spanish version of the Positive and Negative Syndrome Scale (PANSS; Kay et al., 1989). PANSS has been found to be a reasonably valid psychometric tool for people with schizophrenia (Kay et al., 1989; Peralta and Cuesta, 1994), and is one of the most widely used tools for the assessment of therapeutic results in schizophrenia treatment. PANSS was administered to all patients during individualized interviews with the hospital neuropsychologist. It was completed for each patient several times in the month before the study started, during the program and in the month after the end of the program.

The same interview approach was used to assess quality of life, using the EQ-5D (Bobes et al., 2005). The EQ-5D has been found to be reasonably valid for use in people with schizophrenia (König et al., 2007) and is a standard assessment instrument used in this hospital. The neuropsychologist completed the EQ-5D twice with each patient, in the month before the study started and in the month after the end of the program.

Individual attendance at sessions of AAT and the functional program was recorded. Adherence was calculated as the proportion of programmed sessions that a patient attended during the 6-month program period, expressed as a percentage.

In order to study the physiological effects of contact with the dogs during an AAT session, pre- and post-session saliva samples were collected for the last four AAT sessions of the program. Salivary alpha-amylase (sAA) and cortisol were measured. As a biomarker of psychosocial stress, salivary alpha-amylase can be considered to be a measure of the level of activation of the sympathetic nervous system (SNS; Rohleder et al., 2006; Holt-Lunstad et al., 2008). Salivary cortisol is an indicator of the state of the hypothalamic–pituitary–adrenal (HPA) axis and is a general physiological biomarker of stress (Fortunato et al., 2008; Holt-Lunstad et al., 2008). Saliva samples were collected using a commercial saliva collection kit (Salivettes^®^, Sarstedt), with the Salivette remaining in the patient’s mouth for 1 min per sample. Two samples were collected from each patient at each of the four sessions; one was collected 30 min before the AAT session and the other 10 min after the AAT session had finished. Saliva samples were stored in a dry-ice cooled mobile fridge, in which they were delivered to the laboratory to be processed and frozen to −80°C for later testing. The maximum pre-freezing storage time was 4 h. After the study was completed, all saliva samples were thawed and analyzed. Cortisol was extracted and analyzed using a commercial immunoassay (Siemens IMMULITE 2000, Siemens Healthcare Diagnostics. Deerfield, IL, USA; Owen and Roberts, 2011; Tecles et al., 2014), and alpha-amylase was analyzed using a commercial spectrophotometric assay (Olympus AU2700. Olympus America Inc. Center Valley, PA, USA; Tecles et al., 2014).

### Statistical Analysis

We analyzed data from all the participants who completed the 6-month period of the study (*N* = 22). In the present study, patients were included in the analysis regardless of their level of adherence to their medication regime or any of the five elements of the psychosocial rehabilitation process, and adherence to the AAT program was a main outcome measure. As a result, the present study does not comply with the requirements for a ‘per protocol’ analysis, in which patients would be excluded for any deviation from treatment. However, because we excluded two patients who did not complete the study we also did not carry out an ‘intention to treat’ analysis, and so our protocol could be described as a ‘modified intention to treat.’

Between-group (control and AAT-treatment) contrasts of PANSS and EQ-5D scores were analyzed using Statistica 10 and GraphPad Prism 6. Data was tested for normality using the Shapiro–Wilk test; parametric data was tested using a *t*-test, and non-parametric data was tested using the Mann–Whitney *U* (for unpaired data) or Wilcoxon test (for paired data). For dichotomous variables (patient sex), a chi-square test was used to compare proportions between groups. Multiple comparisons were made in the EQ-5D analysis, so the Bonferroni correction was used to adjust the value of *p* that was accepted for significance (for example, for 20 comparisons, *p* = 0.05/20 = 0.0025).

Pre-program PANSS and EQ-5D scores were compared with post-program scores, for the AAT-treatment and control groups separately. After checking normality of data (with the Shapiro–Wilk test), a paired-samples *t*-test was used with parametric data and the Wilcoxon test was used with non-parametric data.

Adherence to treatment data was checked for normality using the Shapiro–Wilk test. An unpaired *t*-test (for parametric data) or Mann–Whitney *U* (for non-parametric data) was used to compare adherence levels between the AAT-treatment group and either overall compliance or compliance for individual activities within the functional programs (control group).

A paired *t*-test was used to compare pre- with post-session levels of cortisol and alpha-amylase in the AAT-treatment group (data had been found to be normally distributed using the Shapiro–Wilk test).

### Ethics

The Clinical Research Ethics Committee of the Hospital del Mar Medical Research Institute (IMIM) approved the clinical-protocol, patient management, and participation of the patients.

The Department of Agriculture and Natural Environment of the Catalonia Government approved the animal management protocol for this study. All dogs that participated in the project were given a thorough medical, behavioral, and welfare assessment before, during, and after the AAT program.

All patients who were eligible for the study received documentation that outlined the study, and they signed an informed consent form. They were able to withdraw from the study at any time.

Animal assisted therapy technicians signed an informed consent form that detailed their responsibilities (confidentiality and conformity) within the project.

Spanish law 15/99 (regarding personal data protection) was applied to all data collection.

## Results

### Sample Characteristics

There were no differences between control and AAT-treatment groups with respect to sex [Chi-square test; χ^2^(1) = 0.40], age or initial scores of PANSS and EQ-5D (Mann–Whitney *U*; *p* < 0.05; See **Table 2** for full details).

**Table 2:** Initial scores of PANSS and EQ-5D of the analyzed patients of this study.

	Mean (*SD*)	
INITIAL SCORES	Treatment (*N* = 14)	Control (*N* = 8)	*U*	*Z* adjusted	2 sided exact *p*^∗^
AGE	48.9 (6.7)	46.7 (7.3)	40.5	1.02	0.29
PANSS positive	18.9 (6.0)	20.7 (5.7)	46	−0.64	0.52
PANSS negative	25.3 (7.5)	21.70 (4.5)	39.5	1.09	0.26
PANSS general	44.3 (12.3)	42.6 (13.7)	51.5	−0.27	0.76
EQ-5D Total score	1.9 (1.8)	1.5 (1.3)	49.5	0.42	0.66
EQ-5D- Health Today (0–100)	80.7 (24.9)	78.7 (18.3)	42	0.94	0.36
EQ-5D- F1 Mobility	0.1 (0.3)	0.1 (0.3)	53	−0.34	0.86
EQ-5D- F2 Personal Care	0.1 (0.4)	^∗∗^	48	1.02	0.61
EQ-5D- F3 Daily Activities	0.1 (0.3)	0.2 (0.7)	52.5	−0.41	0.81
EQ-5D- F4 Pain/Discomfort	0.4 (0.6)	0.5 (0.5)	50	-0.43	0.71
EQ-5D- F6 Health State 12 m	0.4 (0.6)	0.4 (0.7)	51.5	0.33	0.76

*Comparison between control and treatment group. ∗Mann–Whitney U Test. Tests are significant at p < 0.05. No significant results were found. ∗∗Data for the initial item EQ-5D F5 Anxiety was not included because some patients were not able to fully understand the meaning of this item.*

During the program, two patients within the AAT-treatment group withdrew from the study. One patient was discharged from the hospital before the end of the AAT program. The other patient exhibited behaviors that threatened to compromise the welfare of the therapy dogs, and therefore stopped participating in the AAT activity (See **Figure 1**).

### Schizophrenic Symptomatology (PANSS)

At the end of the program, no significant differences were found between control and AAT-treatment groups (Mann–Whitney *U* test, *p* < 0.05) with respect to final PANSS or change in PANSS (see **Table 3** for full details). However, there were significant differences in PANSS pre-treatment and post-treatment scores in both control and AAT-treatment groups (*t*-test; *p* < 0.05). In the AAT-treatment group, scores for all PANSS subscales (positive, negative, and general) were significantly lower after the AAT program (*t*-test; *p* < 0.05). In the control group, only positive and general PANSS scores showed a significant decrease after treatment (*t*-test; *p* < 0.05). For full details, see **Table 4**.

**Table 3:** Differences between control and treatment groups with respect to final PANSS (after 6 months of treatment) or change in PANSS.

	Mean (*SD*)	
	Treatment (*N* = 14)	Control (*N* = 8)	*U*	*Z* adjusted	2 sided exact *p*^∗^
PANSS positive score FINAL	13.6 (3.8)	12.9 (5.2)	52	0.24	0.81
PANSS positive change	5.3 (4.8)	7.9 (4.3)	38.5	1.16	0.23
PANSS negative score FINAL	19.6 (7.0)	19.9 (5.4)	55	−0.03	0.97
PANSS negative change	−11.7 (7.4)	−8.9 (4.8)	41	−0.99	0.33
PANSS general score FINAL	34.3 (8.6)	30.0 (6.0)	37	1.26	0.21
PANSS general score change	5.6 (8.9)	1.9 (3.4)	45	0.71	0.48

*∗Mann–Whitney U Test. Tests are significant at p < 0.05. No significant results were found.*

**Table 4:** Differences in PANSS pre-treatment and post-treatment scores in both control and treatment (AAT) groups.

Group	Variable	Number of pairs (pre vs. post)	Mean (*SD*)	*t* (df)	*p*-value
Control	PANSS positive	8	7.87 (4.29)	*t*(7) = 5.19	0.001^∗^
Control	PANSS negative	8	1.87 (3.44)	*t*(7) = 1.54	0.167
Control	PANSS general	8	12.63 (13.57)	*t*(7) = 2.63	0.033^∗^
AAT	PANSS positive	14	5.28 (4.78)	*t*(13) = 4.13	0.001^∗^
AAT	PANSS negative	14	5.64 (8.19)	*t*(13) = 2.57	0.022^∗^
AAT	PANSS general	14	10.00 (8.70)	*t*(13) = 4.30	0.001^∗^

*∗t-test (considered significant at p < 0.05).*

### Quality of Life (EQ-5D)

No significant difference was found between AAT-treatment and Control groups (Mann–Whitney *U* test; *p* < 0.0025 after Bonferroni correction). In addition, almost none of the EQ-5D items were significantly different after treatment (Wilcoxon test; *p* < 0.05; **Table 5**). Only the score for the general health item (compared with 12 months before) of the EQ-5D was significantly lower after the program in the AAT-treatment group (Wilcoxon test; *p* < 0.05). For this item, low scores indicate higher health status, meaning that AAT-treatment group patients perceived themselves to be in a better state of health after the program. However, after applying a Bonferroni correction none of the results of EQ-5D was significant different after treatment (for eight comparisons, *p* = 0.05/8 = 0.0625).

**Table 5:** Differences in EQ-5D pre-treatment and post-treatment scores in both control and treatment groups.

Group	Variable	Number of pairs	Type of test	*t* (df) or *W*	*p*-value
Control	EQ-5D Total score	8	T	*t*(7) = 1.8	0.11
Control	EQ-5D Health today 12 m	8	W	*W* = 9	0.53
Control	EQ-5D Mobility	8	W	*W* = 0	>0.99
Control	EQ-5D Pain/discomfort	8	W	*W* = 3	0.50
Control	EQ-5D Health State today	8	W	*W* = 0	>0.99
Control	EQ-5D Anxiety/Depression	8	W	*W* = −3	0.50
Control	EQ-5D Daily Activities	8	W	*W* = −1	>0.99
Control	EQ-5D Personal Care	8	^∗∗^	^∗∗^	^∗∗^
Treatment	EQ-5D Total score	14	W	*W* = −3	0.91
Treatment	EQ-5D Health today 12 m	14	W	*W* = 37	0.03^∗^
Treatment	EQ-5D Mobility	14	^∗∗^	^∗∗^	^∗∗^
Treatment	EQ-5D Pain/discomfort	14	W	*W* = −3	0.76
Treatment	EQ-5D Health State today	14	W	*W* = 0	>0.99
Treatment	EQ-5D Anxiety/Depression	14	W	W = 0	0.34		
Treatment	EQ-5D Daily Activities	14	W	*W* = −10	0.07
Treatment	EQ-5D Personal Care	14	W	*W* = 3	0.34

*(T) t-test (data passed normality test). (W) Wilcoxon test (data did not pass normality test). ∗Tests are significant at p < 0.05 (If apply Bonferroni correction: p < 0.05/8 < 0.00625). ∗∗Could not calculate because the values to compare were the same.*

### Adherence to Treatment

**FIGURE 2:**
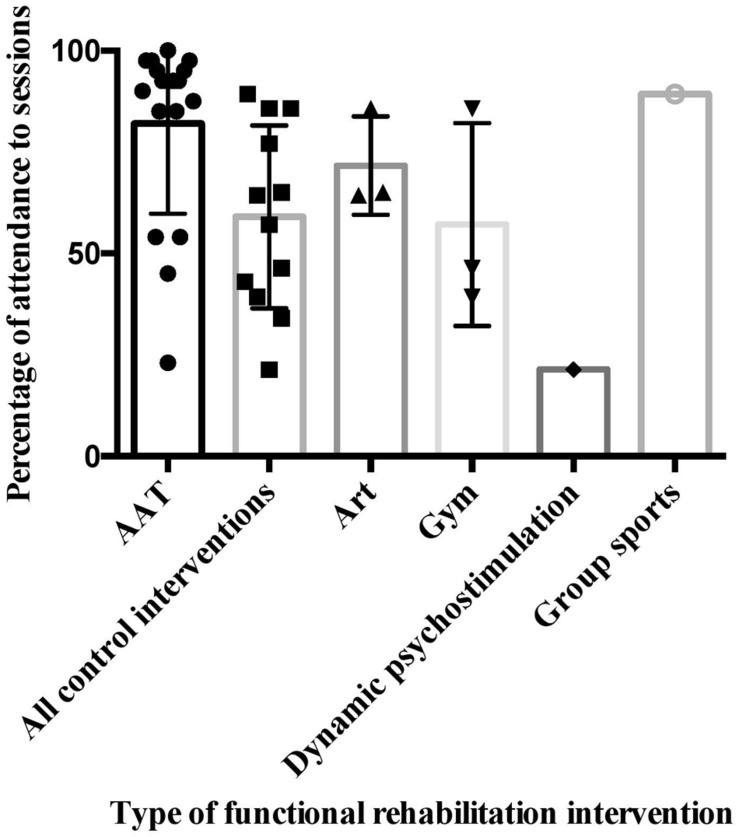
**Differences between AAT and other functional rehabilitation interventions**.

Although patients were encouraged, and expected, to attend all scheduled activities, attendance was entirely voluntary. In the AAT-treatment group, there was an overall 92.9% (*SD* = 4.7) adherence to treatment for the AAT sessions. The majority of absences from the AAT sessions were due to family or health issues. Only once did a patient not want to attend an AAT session. In the control group, there was an overall 61.2% (*SD* = 24.8) adherence to treatment for the assigned activity from the functional program. This higher level of adherence to the AAT sessions, compared with overall adherence to the functional activities, was significant [*t*-test: *t*(20) = 4.7; *p* = 0.0001]. We could only compare adherence to AAT-treatment with specific functional program activities for which the number of attending patients was large enough to justify a statistical test (art therapy and gymnastics). AAT showed significantly better adherence than art therapy (Mann–Whitney *U* test; *U* = 2; *p* = 0.01) and gymnastics therapy (Mann–Whitney *U* test; *U* = 2; *p* = 0.01). All detailed data on adherence to treatment are presented in Tables 6 and 7 and see **Figure 2**.

**Table 6:** Patients’ adherence to treatment.

Patient ID	Group	Number of programmed sessions	Number of attended sessions	Percentage of adherence
1	Treatment	40	38	95
2	Treatment	40	37	92.5
3	Treatment	40	39	97.5
4	Treatment	40	40	100
5	Treatment	40	37	92.5
6	Treatment	40	34	85
7	Treatment	40	39	97.5
8	Treatment	40	36	90
9	Treatment	40	37	92.5
10	Treatment	40	38	95
11	Treatment	40	37	92.5
12	Treatment	40	34	85
13	Treatment	40	39	97.5
14	Treatment	40	35	87.5
15	Control	28	16	57.1
16	Control	28	6	21.4
17	Control	28	13	46.4
18	Control	21	18	85.7
19	Control	28	25	89.3
20	Control	28	18	64.3
21	Control	56	22	39.3
22	Control	56	48	85.7
MEAN(*SD*)	All patients	37.9 (8.4)	31.1 (10.1)	81.3 (21.5)
	Treatment group	40.0 (0.0)	37.1 (1.9)	92.9 (4.7)
	Control group	34.1 (13.7)	20.8 (12.4)	61.2 (24.8)

**Table 7:** Differences in adherence to treatment between AAT and other types of functional rehabilitation interventions.

Type of compared functional intervention	Number of participants in the control group	Type of test	*t* (df) or *U*	*p*
AAT vs. Art therapy	3	U	*U* = 2	0.010^∗^
AAT vs. Gymnastics	3	U	*U* = 2	0.010^∗^
AAT vs. Psychodynamic therapy	1	U	^∗∗^	^∗∗^
AAT vs. Group sport	1	U	^∗∗^	^∗∗^
AAT vs. all other	8	T	*t*(20) = 4.7	0.001^∗^

*(T) t-test (data passed normality test). (U) Mann–Whitney U Test (data did not pass normality test). ∗Tests are considered significant at p < 0.05. ∗∗Not possible to calculate because not enough values in the control type of intervention.*

### Salivary Cortisol and Alpha-Amylase

We collected 61 pre-session and 60 post-session saliva samples from the AAT-treatment group. However, some of the saliva samples were too small for analysis and were discarded. Cortisol analysis was performed with 48 matched pairs of samples (matching every corresponding pre-session and post-session sample for each session for which sufficient sample was available). There was a significant decrease in cortisol after participation in an AAT session (Wilcoxon Test; *p* < 0.05. Pair-matching was confirmed using the Spearman test; *p* < 0.05). Fifty pairs of matching samples were used to measure the effect of the intervention on salivary alpha-amylase. sAA was increased after the AAT sessions, but the difference was not quite significant (Wilcoxon Test; *p* = 0.059. Pair-matching was confirmed using the Spearman test; *p* < 0.0001).

## Discussion

In terms of age and gender, our sample of patients was consistent with the general population of people with schizophrenia, as well as the population of institutionalized people with schizophrenia (Jablensky, 2000; Uggerby et al., 2011). All participants were receiving at least one psychotropic drug, as is common in people treated for this condition (Jablensky, 2000; Uggerby et al., 2011). Our results could therefore be relevant to other similar institutions that are considering the implementation of an AAT program.

With regard to population size, our study was comparable with similar studies that have investigated the effect of AAT in the treatment of schizophrenia, suggesting some common methodological limitations (Barak et al., 2001; Nathans-Barel et al., 2005; Kovács et al., 2006; Berget, 2008; Chu et al., 2009; Villalta-Gil et al., 2009). Apart from the constraint of working with a limited total population of patients within a single hospital unit, and the application of exclusion/inclusion criteria, it should be remembered that AAT has to be conducted in small groups for practical reasons such as the need for proper supervision and a high animal-to-patient ratio (Fine, 2010).

One patient withdrew from the study due to the risk of harm to the therapy dogs. This kind of problem should have been anticipated and taken into account within the exclusion criteria. This should be considered in future studies. Another patient withdrew very early in the study (week 3), and prior to the collection of any outcome data. The recommended approach for superiority studies is an intention to treat analysis, whereby all patients included in the randomization are included in the analysis, and by deviating from this approach in our study we risk an overestimation of the treatment effect (Armijo-Olivo and Magee, 2009). So, whilst the results are interesting and point to a potential effect of treatment, they cannot be relied upon as general evidence of efficacy in a clinical population.

People with a diagnosis of chronic schizophrenia who live in institutionalized settings have very low levels of social functioning and social activity (Kovács et al., 2004). Individual or combined measures of symptomatology, quality of life and adherence to treatment are commonly used to assess the efficacy of a psychosocial rehabilitation process for patients with schizophrenia (Wilson-d’Almeida et al., 2013), but not together in the same study. By including these measures and adding an assessment of salivary cortisol and alpha-amylase, our study provides an interesting insight into the use of combined measures.

In terms of symptomatology, in the AAT-treatment group we observed an improvement in negative symptoms of schizophrenia like apathy, asociality, anhedonia and alogia, that could be partially explained by the regular interaction between patients and animals. Previous work suggests that AAT programs may be effective in the control of negative symptoms of schizophrenia (Barker and Dawson, 1998; Barak et al., 2001; Kovács et al., 2004; Nathans-Barel et al., 2005). Therapy dogs have been described as social catalysts or mediators of interactions between patients and between patients and their therapists, and these benefits could be extended outside the AAT sessions (Fine, 2010). Since negative symptoms of schizophrenia are relatively insensitive to pharmacological therapies and are associated with a chronic course and high levels of social disability, it is very important to find effective alternative interventions that can be added to standard treatment protocols (Hammer et al., 1995; Liddle, 2000; Gråwe and Levander, 2001). The beneficial effects of AAT on negative symptoms of schizophrenia is therefore worthy of further investigation.

The trend toward an increase in alpha-amylase combined with the significant decrease in cortisol after the AAT sessions suggests that the interaction patients had with the dogs was perceived to be not only engaging, but also relaxing. Increases in alpha-amylase and the activation of the SNS can occur in positive emotional states (Fortunato et al., 2008; Payne et al., 2014), and recent research indicates that people with schizophrenia may experience a dysregulation of SNS tone (Monteleone et al., 2015).

The lack of significance for the change in salivary alpha-amylase could be due to the absence of an effect, but also due to the small population size and the small number of collected saliva samples (saliva was only collected for the last four AAT sessions, sample collection was not always successful, and approximately 17% of collected samples had to be rejected due to inadequate sample volume for analysis).

Regarding stress and cortisol levels, previous research has found decreases in salivary cortisol during AAT sessions in other types of patients, such as autistic children (Viau et al., 2010) and insecure attached males (Beetz et al., 2012a). In a previous study with people being treated for schizophrenia, cortisol levels were not been found to change after interaction with animals (Nepps et al., 2014). However, in comparison to our study, the AAT protocol for that study did not include repeated sessions for each patient and the ratio of dogs per patient was lower. Long-term and dose effects of AAT on stress levels of patients with schizophrenia still need to be studied. Future studies could take advantage of our experience by extending the measurement of salivary cortisol to all AAT sessions within a program, and a control group, while also looking for long-term and dose effects.

There were some difficulties in collecting saliva samples in this study, both in terms of quantity and quality of saliva. The pharmaceutical treatment of schizophrenia involves drugs that suppress salivation, and as a consequence of their symptomatology, many people with schizophrenia are smokers (Rae et al., 2014). Smoking increases cortisol and decreases alpha-amylase (Granger et al., 2007a), so this could be a confounding factor. Future studies should include data on patients’ smoking level, particularly when comparing saliva measures between groups, as between group matching could be important. In addition, personal hygiene and dental care seems to be poor in many people with schizophrenia (Velligan et al., 1997), and the presence of impurities in saliva samples could interfere with the reliability of the measurements (Granger et al., 2007b). Ideally, a patient should have rinsed his or her mouth with water some minutes before saliva collection, but due to a lack of patient cooperation this was rarely possible. Future research should try to extend and optimize saliva sample extraction and analysis, as it seems cortisol and alpha-amylase could be good markers of AAT effects in people being treated for schizophrenia.

Quality of life measurements did not differ between pre-treatment and post-treatment conditions in either of the two groups. Improvement in symptomatology is not always related to improvement in quality of life in people with schizophrenia as the latter can be affected by other factors such us the level of insight (Wilson-d’Almeida et al., 2013; Hayhurst et al., 2014; Margariti et al., 2015). Previous research has shown that even patients with schizophrenia who are undergoing treatment can experience a progressive decline in their quality of life (Medici et al., 2015). Therefore, a lack of decline in overall quality of life measurements could be interpreted to be a benefit of psychosocial rehabilitation, particularly in chronic patients. Future research could focus on specific domains of quality of life where AAT seems to have a direct effect, such as anxiety and depression (Barker and Dawson, 1998) and social relationships (Villalta-Gil et al., 2009).

In the present study, mean adherence to the alternative functional rehabilitation interventions (art therapy, group sports, dynamic psycho-stimulation, or gymnastics) was lower in the control group than the AAT-treatment. Previously reported adherence rates to therapeutic sport programs for people being treated for schizophrenia range from 50 to 82% (Beebe et al., 2005; Warren et al., 2011). In the present study, there were intrinsic differences between the activities included in the functional program, but they all shared certain features, such as frequency, duration, and being conducted outside the hospital. Although the added value of AAT sessions in terms of adherence could be due to a novelty effect, attendance to sessions did not decline during the program. Information about adherence is rarely reported in AAT research, but it could be a very useful indicator in the context of psychosocial rehabilitation, and deserves further research (Kamioka et al., 2014).

Another factor that could be of importance in adherence to ATT is the human–dog relationship (Nagasawa et al., 2015). An initial bond may be quickly established between a person and a dog, and this bond has a strongly emotional element (Dwyer et al., 2006; Fine, 2010; Beetz et al., 2012b), that leads to the development of attachment to the dog (Zasloff, 1996). This attachment could contribute to a person’s sustained interest in attending AAT sessions, but could potentially lead to problems when the human–animal bond is disrupted at the end of the program. Further research could monitor the development of the patient–dog bond during an AAT program, and the effects of ending such programs.

Taken together, the various significant results reported in this study (reduction of negative symptomatology, high adherence to the AAT program, and cortisol reduction after AAT sessions) could be explained by the biology of human–animal interactions (Beetz et al., 2012b; Nagasawa et al., 2015). When a person has a enjoyable contact with a dog there is a release of oxytocin, dopamine, and endorphins, as well as a decrease in cortisol (Beetz et al., 2012a,b; Julius et al., 2013). This overall reaction seems to enhance pro-social behavior and decrease anxiety and stress, mainly via the hypothalamic-pituitary axis (HPA; Neumann et al., 2000). Oxytocin administration has previously been proposed as a treatment for psychiatric patients because of its broad pro-social effects on behavior and cognition (Zik and Roberts, 2014). Through the release of oxytocin, positive contact with dogs could produce such psychosocial and psychophysiological benefits. Future research in AAT might also try to study changes in oxytocin levels of people being treated for schizophrenia during contact with animals.

The results of our study raise some questions that could be addressed in future work. Adherence to treatment is a significant problem, especially in lengthy rehabilitation programs with challenging patients. It would be interesting to investigate whether the high level of adherence to AAT that we observed is replicated in other therapeutic situations, and whether adherence really is different from other closely matched activities. It is possible that the mere presence of a dog in any type of therapy session could improve adherence, especially if the patient has developed a relationship with the dog during AAT, and this effect should be investigated. In all rehabilitation programs resources are limited and the inclusion of AAT could represent an opportunity cost by displacing other activities. It is therefore important to find out whether patients who have participated in AAT go on to experience significant long-term benefits after the rehabilitation program has concluded, compared with patients who have been involved in other activities.

## Conclusion

Animal assisted therapy seems to be a worthwhile adjunct therapeutic approach for people being treated for schizophrenia in a conventional psychosocial rehabilitation process, with potential positive outcomes in symptomatology, adherence to AAT program, and stress reduction during AAT sessions.

## Author Contributions

The paper itself was written by PC, JF, and JB. The paper was reviewed before submission by AB, JRF, SG, CM, OO, FM, AT, JC, and MG. All authors contributed to the initiation and design of the study. PC, JF, JRF, SG, CM, MG, and AB monitored the progress of the study. PC, JF, and JB decided on the analytic strategy. JB, JRF, SG and CM equally contributed to the total production of the study. PC is the guarantor of the study.

## Conflict of Interest Statement

The Chair Affinity Foundation Animals and Health is sponsored by a non-profit Foundation (Affinity Foundation). Any research The Chair Affinity Foundation Animals and Health develops is not related to any commercial product.
